# Assessing the Use of Mobile Health Technology by Patients: An Observational Study in Primary Care Clinics

**DOI:** 10.2196/mhealth.4928

**Published:** 2016-04-19

**Authors:** Veronica Ramirez, Emily Johnson, Cesar Gonzalez, Vanessa Ramirez, Barbara Rubino, Gina Rossetti

**Affiliations:** ^1^ Department of Internal Medicine Los Angeles County + University of Southern California Medical Center Los Angeles, CA United States; ^2^ Keck School of Medicine University of Southern California Los Angeles, CA United States

**Keywords:** Mobile health applications, mobile health technology, telemedicine

## Abstract

**Background:**

There is significant potential for mobile health technology to improve health outcomes for patients with chronic diseases. However, there is a need for further development of mobile health technology that would help to improve the health of lower-income communities.

**Objective:**

The study objective was to assess mobile phone and app usage among a culturally diverse patient population, and to determine whether patients would be interested in using mobile health technology to help manage their chronic diseases.

**Methods:**

An observational study was conducted with patients of the Internal Medicine resident primary care clinics of Los Angeles County and University of Southern California (LAC+USC) Medical Center. Self-reported information regarding demographics, current mobile phone usage, current mobile health app and social media usage, barriers to using mobile phones or mobile health apps, and interest in using a mobile health app was collected.

**Results:**

Ninety-one percent of patients owned a mobile phone, with 76% (169/223) of these reporting having a mobile phone with Internet capability. Fifty-seven percent of subjects used mobile apps on their mobile phones, and 32% (41/130) of these used mobile apps related to their health. Eighty-six percent (207/241) of respondents voiced interest in using a mobile app to improve their health, and 40% (88/221) stated they would use such an app daily. Patients stated they would find the mobile health app most useful for nutrition, exercise, and obtaining general information on medical conditions.

**Conclusions:**

Despite the fact that the majority of our primary care patients were of lower socioeconomic status, they utilized mobile phones with Internet and mobile app capabilities to a great extent. There was substantial interest among our patients in using mobile health technology to both manage chronic disease and improve overall health. Given that cultural, educational, and socioeconomic disparities strongly correlate with higher rates of chronic diseases such as obesity, diabetes and hypertension, access to culturally relevant mobile health tools may empower patients in these populations to improve health outcomes.

## Introduction

Mobile phones have developed into more than just a convenient way to place a phone call. These devices have become tools necessary to complete such tasks as managing a schedule, checking urgent work emails, and even keeping track of physical activity. In 2012, 85% of American adults owned some type of mobile phone [ 1]. In 2014, 64% of American adults owned a smartphone [ 2]. For the health professional, a mobile phone has become an integral part of the delivery of health care. Mobile health technology, or simply, *mHealth*, has experienced incredible growth in the last ten years with the widespread adoption of smartphones. The principle components of *mHealth*, such as text messages (SMS) and mobile health apps, give health providers quick access to medical information in order to help care for their patients. In return, patients have utilized *mHealth*to take more active roles in managing their own health. In 2012, 31% of mobile phone owners reported using their phone to search for health or medical information on the Internet, and 19% of mobile phone owners reported using mobile health apps to track and manage their health [ 1]. In a descriptive analysis of “health and wellness” apps available on the Apple iTunes Store in 2014, the most popular mobile health apps focused on self-monitoring of exercise, diet, and weight management [ 3], indicating that developers see a market in individuals interested in taking a more proactive role in managing their health.

Though mobile health apps continue to be researched and developed, the role of *mHealth*in chronic disease management in patients that belong to culturally, linguistically, and economically diverse communities remains an area of ongoing work. Historically, a “digital divide” has existed in which individuals belonging to such communities lack access to digital information (especially Internet access) because of their particular demographics [ 4, 5]. However, several studies have shown promise that the digital divide may be gradually closing as the Internet and mobile technology become more readily accessible to underserved populations. In the 2013 Hispanic Trends Project of the Pew Research Center, it was observed that Latinos owned mobile phones and went on the Internet from mobile devices at similar, and even sometimes higher, rates than did other groups of Americans, and it was very likely these trends would continue to increase [ 6]. Therefore, there needs to be further research into how such populations, who historically suffer from significant health disparities, may benefit from the development of culturally and linguistically tailored mobile health technology.

The purpose of this study was to investigate mobile phone usage among patients at the Los Angeles County and University of Southern California (LAC+USC) resident primary care clinics, which serve a diverse patient population. We aimed to assess how patients utilize various types of mobile technology, and to what extent our patients use mHealth. We also wanted to assess need for and interest in using mobile health technology, such as mobile phone apps and social media, to help manage health (for example, obtaining information regarding nutrition, physical activity, and chronic disease management). As a secondary aim, we were interested to determine whether demographic factors predicted engagement with social media and mobile phone apps, given that there is expanding growth in reaching these patients via these mobile health platforms.

## Methods

### Survey Development

The study was approved by the Internal Review Board of the University of Southern California. An electronic survey of 25 questions was designed by a team of general internists familiar with the target patient population. Based on a literature search of research surveys regarding mobile health [ 1- 2, 5], questions were selected and modified for our patient population. The survey assessed several categories (the full survey is available in Textbox 1).

**The 25-question electronic survey.**

1. What is your age?

2. What is your sex?

3. What is your ethnicity?

4. What is your primary language?

5. What other languages do you speak?

6. What is your annual household income?

7. What is your highest level of education?

8. Do you own a cellular phone?

9. If yes to #8, what kind of cellphone are you using (with or without internet capability)?

10. If your cellphone has internet capability, do you use the internet?

11. If you use the internet on your cellphone for your health needs, what website do you use?

12. Do you know what the definition of a cellphone application (app) is? If so, please explain.

13. Do you use your cellphone to access applications?

14. If yes to #13, do you use applications related to your health?

15. Please list the cellphone applications related to your health that you use on a regular basis.

16. If you do not have a cellular phone that accesses the Internet/applications, do you know someone who does?

17. If you identified someone in #16, do they use their cellular phone to access the internet/applications for your health needs?

18. Do you own a social media account? Please click all that apply.

19. How often do you log into social media networks?

20. What are the barriers to using the Internet/applications on your phone or using social media?

21. Would you be interested in using social media to network with other people with similar health issues?

22. Would you be interested in using a cellphone application to improve your health?

23. If yes to #22, how often would you use it to improve your health?

24. What particular cellphone health application would be useful to you (you may choose more than one)?

- nutrition information

- exercise tracker

- calorie counter

- general health information on chronic diseases

- mental health

- glucose log for diabetes management

25. When you access social media or applications on your cellphone, what is the primary language of the interface?

#### Demographic Information

Demographic information included age, gender, race/ethnicity, primary language spoken, annual household income, and highest level of education achieved. After the initiation of data collection, it was noted that the income categories used (consistent with other studies and census categories) may not have fully captured the socioeconomic background of our patient population, which has a large proportion of uninsured and undocumented persons. Therefore, a demographic question with regard to education level was added to the final survey after 65 patients had already been interviewed. After this question was added, no further changes were made to the final study survey.

#### Mobile Phone Usage

Participants were asked if 1) they owned a mobile phone, 2) the mobile phone they owned had Internet capability, and 3) they used the Internet on their mobile phone to learn about their health. Afterwards, the participants were asked about their knowledge of mobile phone apps, if they used such apps, and if they were currently using any mobile health apps. In addition, we asked participants to identify individuals who they might be relying on to use mobile phones and/or mobile apps for them (eg, a partner, child, or friend).

#### Social Media Usage

We asked participants about current use of social media networks, such as Facebook, Twitter, and Instagram, and to what extent they used these networks.

#### Interest in Mobile Health Apps and Social Media

Participants were asked if they were interested in using social media to connect with other patients in the same clinic. They were then asked about their interest in using mobile health apps to help improve their health, how often they would use such an app, and what type of app would be most useful to them (eg, obtaining nutrition information, an exercise log, information about chronic diseases).

### Participants and Setting

All participants were patients of the 3 primary care clinics staffed by the Internal Medicine residents at LAC+USC Medical Center. In total, LAC+USC Medical Center serves close to 60,000 primary care patients empaneled under the Los Angeles County Department of Human Services. Historically, these clinics have served indigent, uninsured patients of Los Angeles County. Seventy-five percent of the LAC+USC patient population identifies as Hispanic/Latino. With the implementation of the Affordable Care Act, many patients now have state-based exchange health insurance plans. However, these clinics continue to be safety nets, serving undocumented and uninsured patients. Given the nature of these safety net clinics, patients are advised to arrive at least a half hour prior to their scheduled appointment. Patients may wait to be seen for as long as a few hours, which allowed us to recruit a large sample of potential participants.

### Survey Delivery

Patients were invited to participate from the general medicine clinic waiting room, and allowed to participate if they had sufficient time to complete the entire survey, solely based on their upcoming appointment time. Bilingual research assistants acquired verbal informed consent and offered assistance to participants while they completed the survey on a provided iPad. Certified medical interpreters were used to interpret the survey (via telephone) for patients whose primary language was not English or Spanish, including Mandarin, Cantonese, and Tagalog. Patients were not provided with remuneration.

## Results

A total of 244 adult patients agreed to participate and completed the survey from June 2014 to March 2015. Interviewers reported an average 10% nonparticipation rate among patients with time to complete the survey. Table 1summarizes the demographic information of the sample. Of these subjects, 234/244 (96%) were under the age of 65, 139/244 (59%) were female, 184/244 (75%) identified themselves as Hispanic or Latino, and 135/244 (55%) identified Spanish as their primary language. Furthermore, 172/217 (79%) reported an annual household income of less than $20,000, 113/178 (63%) had earned at least a high school diploma, and 40/178 (23%) had earned a college or professional degree.

Table 2highlights the findings regarding mobile phone usage. The great majority of participants (91%, 223/244) owned a mobile phone. Of these 223 subjects, 169 (75%) owned a phone with Internet capability and 136 (72%) used the internet on their phone on a regular basis. Seventy-six percent of Internet phone users reported using the Internet on their mobile phone to search for information regarding their health. The most common websites used to search for health information were 1) Google, 2) WebMD, and 3) YouTube. In terms of mobile phone apps, 67% (160/238) of participants reported knowing the definition of a mobile app. Fifty-seven percent (130/227) reported using apps on their mobile phone, and of these participants, 41/130 (31.5%) used mobile phone apps related to their health. With regard to social media usage, 134/244 (55%) used social media, and 57% (91/160) reported using social media at least once a day. The most common social media platforms reported were Facebook (51%, 125/244) and Twitter (13%, 31/244).

**Table 1:** Participant demographics (n=244).

Variable		n (%)
**Age**	18-24	20 (8.2)
	25-34	29 (11.9)
	35-44	48(19.7)
	45-54	75 (30.7)
	55-64	62 (25.4)
	65-74	9 (3.7)
	75+	1 (0.4)
**Sex**	Female	139 (58.6)
	Male	98 (41.4)
**Race/Ethnicity**	Mixed Race, Other Race	3 (1.2)
	Asian or Pacific Islander	20 (8.2)
	Black or African American	19 (7.8)
	Hispanic or Latino	184 (75.4)
	White/Caucasian	16 (6.6)
	Prefer not to answer	2 (0.8)
**Primary Language**	Spanish	135 (55.3)
	English	96 (39.3)
	Mandarin/Cantonese	2 (0.8)
	Tagalog	6 (2.5)
	Other	5 (2.1)
**Second Language**	English	77 (51)
	Spanish	65 (43)
	Other	10 (6)
**Annual Household Income (US dollars, n=217)**	< 20,000	172 (79.3)
	20,000-40,000	33 (15.2)
	40,000-60,000	5 (2.3)
	60,000-80,000	4 (1.8)
	80,000-100,000	1 (0.5)
	> 100,000	2 (0.9)
**Education Level (n=178)**	No schooling	3 (1.7)
	Elementary or Middle School	33 (18.5)
	Some High School	29 (16.3)
	High School Diploma	37 (20.8)
	Some College	36 (20.2)
	College Degree	29 (16.3)
	Masters/Professional	11 (6.2)

**Table 2:** Mobile phone/Internet usage

Item		n (%)
**Do you have a mobile phone?**	Yes	223 (91.4)
	Yes, with Internet capability	169 (75.8)
	Yes, without Internet capability	55 (24.7)
	No	21 (8.6)
**Do you have a social media account?**	Facebook	125 (51.2)
	Twitter	31 (12.7)
	Google Plus	26 (10.7)
	Instagram	44 (18.0)
	Myspace	2 (0.8)
	Other	2 (0.8)
**How often do you log into social media networks?**	Less than a few times a month	34 (21.3)
	A few times a month	15 (9.4)
	A few times a week	20 (12.5)
	About once a day	44 (29.4)
	More than once a day	47 (27.5)
**When you access social media or apps on your mobile phone, what is the primary language of the interface?**	Spanish	107 (49.3)
	English	110 (50.7)
**Do you know the definition of a mobile phone “App?”**	Yes	160 (67.2)
	No	71 (29.8)
	Not sure	7 (2.9)
		
**Do you use apps on your mobile phone?**	Yes	130 (57.3)
	Yes, and use health-related apps	41 (31.5)
	No	97 (42.7)
**If you use the Internet on your mobile phone for health needs, which sites do you use?**	Google	45 (33)
	WebMD	15 (11)
	YouTube	7 (5)
	Yahoo/Bing/Other Search Engine	3 (2)
	Mayo Clinic/PubMed/NIH	2 (1)
	Other	7 (5)

**Table 3:** Interest in health apps/social media.

Item		n (%)
**Would you be interested in using a mobile phone app to improve your health?**	Yes	207 (85.9)
	No	34 (14.1)
**If you are interested in using a health app, how often would you use it to improve your health?**	Every day	88 (39.8)
	Every week	83 (37.6)
	Every month	30 (13.6)
	Every 2-3 months	6 (2.7)
	Not at all	14 (6.3)
**What particular health app would be useful to you?**	Sugar log for diabetes	84 (34.4)
	Nutrition information	134 (54.9)
	Calorie counter	81 (33.2)
	Exercise log/tips	107 (43.9)
	Mental wellness	62 (25.4)
	General information about diseases	130 (53.3)
	Other	23 (9.4)
**Would you be interested in using social media to network with other people with similar health issues?**	Yes	168 (70.3)
	No	71 (29.7)

Table 3highlights the findings from the assessment of subjects interested in using mobile health technology and social media. A total of 207/241 (86%) participants were interested in using a mobile phone app to improve their health, and 40% (88/207) participants stated they would use such an app on a daily basis. These participants most often expressed an interest in using a mobile health app that allowed them to search for general information about various medical conditions as well as exercise logging and nutrition information. In addition, 70% (168/239) of participants stated they would use a social media network to connect with other patients who had similar health problems.

In order to explore predictors of social media and mobile phone app engagement, we examined patients’ current use of these platforms. Adjustments were made for broad, commonly used demographic factors that we felt may have influenced mobile phone use. In a multivariate logistic regression model adjusted for phone language, ethnicity, age, income, sex, and education ( Table 4), having a phone interface in Spanish was associated with 63% reduced odds of using social media (95% CI 0.14-0.94), and 85% decreased odds of using health-related apps (95% CI 0.03-0.68). Participants under 55 years old were nearly three times more likely to use social media (OR 2.43, 95% CI 1.05-5.66) but were not significantly more likely to use health apps. No significant interaction was found between phone language and ethnicity.

**Table 4:** Multivariate models to predict health app and social media use among participants with mobile phones.

Model Outcome		OR	95% CI
**Use Health Apps (n=120)**	Phone language (Spanish vs English)	0.15	(0.03-0.68)
	Hispanic, yes or no	0.86	(0.30-2.43)
	Age under 55	1.37	(0.43-4.34)
	Income under $20,000	1.26	(0.41-3.90)
	Sex	0.46	(0.17-1.17)
	High school degree or above	0.81	(0.19-3.37)
**Social Media Use (n=154)**	Phone language (Spanish vs English)	0.37	(0.14-0.94)
	Hispanic, yes or no	0.50	(0.17-1.49)
	Age under 55	2.43	(1.05-5.66)
	Income under $20,000	0.95	(0.38-2.38)
	Sex	0.55	(0.26-1.14)
	High school degree or above	1.23	(0.50-3.01)

## Discussion

Our study highlights several important findings that have implications with regard to improving the health of patient populations that historically have suffered from health disparities. We found that the vast majority of our primary care clinic patients, despite being of lower socioeconomic status and coming from diverse cultural and linguistic backgrounds, owned mobile phones with advanced Internet and mobile app capabilities. These findings are consistent with several studies that have found an increased use of mobile phones with Internet capability among populations of great cultural, educational, linguistic, and economic diversity [ 1, 7- 8]. Furthermore, specific populations, such as Americans with low household incomes and education levels, nonwhites, and females, are much more dependent on mobile phones for accessing the Internet to search for information regarding their health [ 8- 10]. In our population, we found that 41/165 (25%) participants responding to questions about app use endorsed using mobile health apps compared to 19% nationally [ 1]. Given this comparable usage of mobile internet technology by these groups, further development of culturally and linguistically relevant mobile health apps should be pursued.

A majority of study participants relied primarily on search engines such as Google and Yahoo to search for topics or certain health questions related to their own health. This appeared to be the simplest method of initiating an Internet search on specific health issues for our patients. In addition, a few participants identified YouTube as an effective tool for learning about their health. One subject in particular found that the visual/audio features were useful for learning about health topics, and watching such videos made her feel like she was receiving individualized education about that specific health topic. Prior research has shown that visual aids are effective methods of educating patients of lower health literacy [ 11, 12], and the addition of audio instruction may be even more beneficial to patients with limited educational backgrounds. Although this was not a particular focus of our study, this does provide an opportunity for future research to assess the interest and use of online instructional and educational health videos by medically underserved patients.

Over half of participants who owned a mobile phone in our study reported regular use of mobile phone apps. Thirty-one percent of these participants actually used apps related to their own health management. These findings reflect a rise in mobile health app usage among ethnically diverse populations; in 2014, only 15% of Hispanics reported use of health-related mobile phone apps [ 2]. It is becoming apparent that these culturally diverse patient populations are realizing the utility of mobile health apps in helping to manage their own health.

In general, a great majority of our study participants (86%, 207/241) stated they were interested in using a mobile health app to learn more about their health and how to manage their chronic diseases. Further, our patients reported they would use such an app on a regular basis, showing that patients were interested in taking initiative to monitor their own health. Most patients were interested in tools that would help educate them about chronic diseases and good nutrition, and in monitoring and advice tools regarding physical activity. Mobile health technology could address barriers to health care that these groups face, including language barriers preventing health education as well as limited access to care due to lack of resources in safety net clinics [ 13]. Patients reported that they would prefer to use apps in their primary language (Spanish being the language most often identified). Our study shows that there is a high demand for development of such technology and efforts should aim to provide these particular patients with these tools.

In addition to using mobile health apps, a large number of patients reported interest in using a social media network to connect with other patients if this resource were made available. Prior research has shown how social networks, such as Facebook and Twitter, have been effective in improving outcomes in patients with chronic diseases such as HIV/AIDS, breast cancer, and chronic tobacco use [ 14- 16]. These studies have shown that patients using social media networks are willing to share their health information in order to educate others about disease awareness and treatment, as well as to ask questions of other patients who may be suffering from similar symptoms and undergoing similar treatments [ 17- 20]. Our study shows that our diverse clinic patient population is interested in using social networking to meet other patients with similar health issues, which could encourage patients to build a support network for themselves and increase motivation and social support to make positive changes.

In terms of limitations of our study, the number of patients who completed the survey was fairly small. This was, by definition, a convenience sample. However, all efforts were made to recruit as many participants as possible and facilitate completion of the survey with the available interpreters. As mentioned previously in the methods section, a question with regard to education level was not added to the final survey until after 65 patients had already completed the survey. This, and the ability to skip questions, unfortunately resulted in a smaller sample size being used for our secondary analysis ( Table 4). However, because all other methodology of the survey collection, including clinic location, survey collection hours, and languages spoken, remained unchanged, we do not feel these patients are substantively different from our study population as a whole. Furthermore, though we assessed current use of mobile health apps, we did not specifically explore ways our patients used social media with regard to their health. Finally, due to the lack of standardized instruments regarding this research topic, we were unable to statistically validate the intervention questions in our survey tool. However, all survey questions were consistent with previously published literature in the field.

### Conclusion

Our study explored important issues with regard to *mHealth*use by culturally, linguistically, and educationally diverse patient communities. Though the majority of our primary care patients are of lower socioeconomic status, our patients utilize mobile phones with Internet and mobile app capabilities to a great extent. There is substantial interest among our patients in using mobile health technology, including mobile health apps and social networking resources. Given that cultural, educational, and socioeconomic disparities strongly correlate with higher rates of chronic diseases such as diabetes and hypertension, access to culturally relevant mobile health tools may help both patients and providers with management of these conditions. More research is needed to investigate the specific health needs of these patient populations in order to guide development of culturally and linguistically tailored mobile health technology.

## Abbreviations

mHealth: mobile health
LAC+USC: Los Angeles County + University of Southern California
